# Supplementary material for: Effects of Manganese Porphyrins on Cellular Sulfur Metabolism
Source: Molecules. 2020 Feb 22;25(4):980. doi: 10.3390/molecules25040980 (PMC7070779; doi:10.3390/molecules25040980)
Supplement: Supplementary file 1 [file molecules-25-00980-s001.pdf]

Supplementary material

Effects of Manganese Porphyrins on Cellular Sulfur Metabolism

Kenneth R. Olson[a,b], Yan Gao[a], Andrea K. Steiger[c], Michael D. Pluth[c], Charles R. Tessier[a], Troy A. Markel[d], David Boone[a], Robert V. Stahelin[e], Ines Batinic-Haberle[f], and Karl D. Straub[g,h]

a. Indiana University School of Medicine - South Bend Center, South Bend, Indiana 46617 USA

b. Department of Biological Sciences, University of Notre Dame, Notre Dame IN 46556

c. Department of Chemistry and Biochemistry, University of Oregon, Eugene, OR 97403

d. Indiana University School of Medicine, Riley Hospital for Children at IU Health, 705 Riley Hospital Dr, RI 2500, Indianapolis, IN 46202

e. Department of Medicinal Chemistry and Molecular Pharmacology, Purdue University, West Lafayette, IN 47907

f. Department of Radiation Oncology, School of Medicine, Duke University, Durham, NC 27710

g. Central Arkansas Veteran's Healthcare System, Little Rock, AR 72205

h. Departments of Medicine and Biochemistry, University of Arkansas for Medical Sciences, Little Rock, AR 72202

Running Head: Cellular Sulfide Metabolism by MnPs

Address correspondence to:

Kenneth R. Olson, Ph.D.
Indiana University School of Medicine -South Bend
Raclin Carmichael Hall
1234 Notre Dame Avenue
South Bend, IN 46617
Phone: (574) 631-7560
Fax: (574) 631-7821
e-mail: olson.1@nd.edu

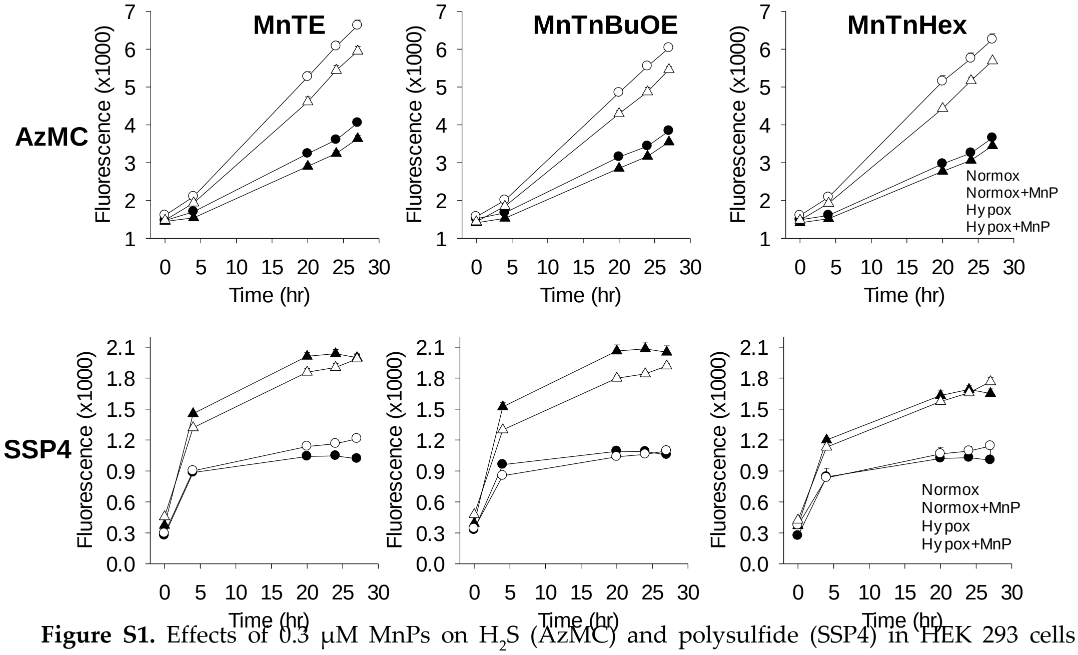

Figure S1. Effects of 0.3 μM MnPs on H_2S (AzMC) and polysulfide (SSP4) in HEK 293 cells in hyperoxia (21% O_2, solid symbols) and physioxia (5% O_2, open symbols). Intracellular H_2S and polysulfides were increased in physioxia compared to hyperoxia, whereas the effects of MnPs were minimal in both conditions. Mean +SE, n=8 wells per treatment.

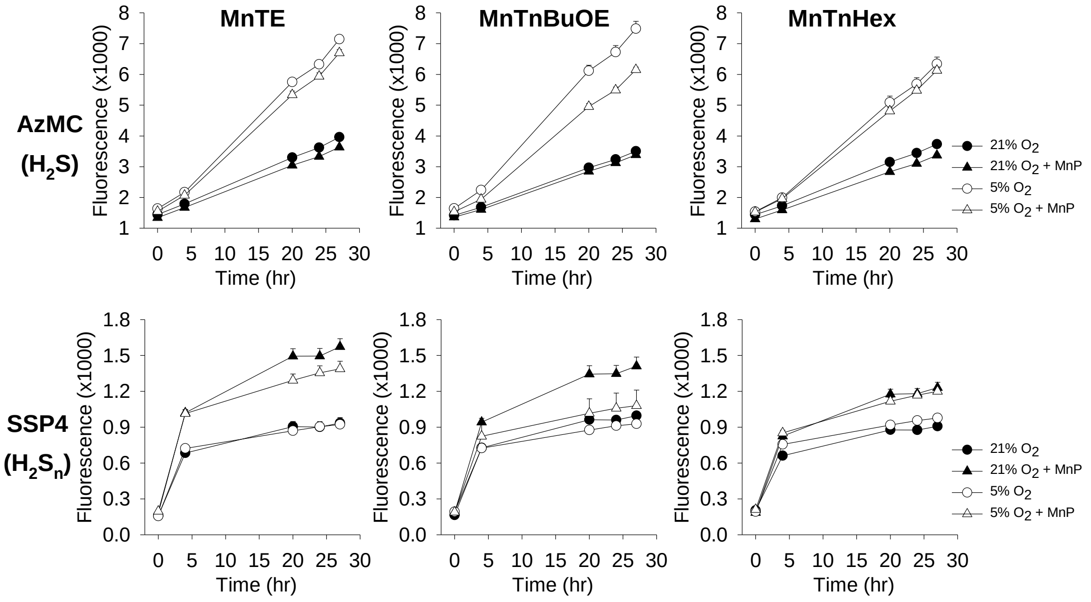

Figure S2. Effects of 0.03 μM MnPs on H_2S (AzMC) and polysulfide (SSP4) in HEK 293 cells in hyperoxia (21% O_2, solid symbols) and physoxia (5% O_2, open symbols). Intracellular H_2S and polysulfides were increased in physioxia compared to hyperoxia. All MnPs decreased intracellular H_2S and increased intracellular polysulfides in both normoxia and hypoxia. Mean +SE, n=8 wells per treatment.
